# Hemothorax due to Ruptured Mycotic Aneurysm of Intercostal Arteries Associated with Infective Endocarditis

**DOI:** 10.1155/2017/9213514

**Published:** 2017-10-31

**Authors:** Eddie Y. Liu, Jennifer Crawford, Haissam Haddad

**Affiliations:** ^1^Department of Internal Medicine, University of Saskatchewan College of Medicine, Saskatoon, SK, Canada S7N 5E5; ^2^Regina Qu'Appelle Health Region, 2180-23rd Avenue, Regina, SK, Canada S4S 0A5

## Abstract

We present a case of hemothorax due to ruptured mycotic aneurysm in three intercostal arteries in a 40-year-old male with methicillin-resistant *Staphylococcus aureus* infective endocarditis (IE) due to intravenous drug use. Microcoil embolization and thoracotomy successfully achieved hemostasis. Mycotic aneurysm is a rare complication of IE and is usually found in the intracranial vessels. Ruptured mycotic aneurysm in the intercostal arteries can be associated with IE and can present as acute hemothorax.

## 1. Introduction

Infective endocarditis (IE) is an infection of the endocardium caused by a variety of microorganisms and may readily extend to involve the valves and adjoining structures of the heart. It is reported that the incidence of IE is estimated to be 3 to 7 cases per 100,000 persons in developed countries. Although uncommon, IE is often a fatal pathologic condition with a mortality rate of 25% [1].

## 2. Case Presentation

A 40-year-old Caucasian man with active intravenous (IV) cocaine use presented with a three-day history of increasing right shoulder pain and a ten-day history of low-grade fever. He had no other past medical history. His only regular medication was methadone 200 mg PO daily. Two separate blood cultures were positive for methicillin-resistant *Staphylococcus aureus* (MRSA). Transesophageal echocardiogram was normal; however, CT scan of the chest and right arm demonstrated myositis of right shoulder and bilateral cavitating lung lesions in keeping with pulmonary septic emboli. MRSA bacteremia with pulmonary emboli strongly suggested a diagnosis of right-sided endocarditis, with any vegetation likely embolized to the pulmonary circulation, thus explaining the normal echocardiogram. This diagnosis was also supported by Duke criteria. He was treated with vancomycin IV at 15 mg/kg/dose q12h to obtain a serum trough concentration of 15 to 20 mcg/mL and rifampin 300 mg PO q8h. An ultrasound of the right arm revealed thrombosis in basilic and brachial veins, and he was started on enoxaparin 1 mg/kg Subq twice a day for treatment of suppurative thrombophlebitis. Blood cultures after 5 days of antibiotic therapy showed no bacteremia.

**Figure 1:**
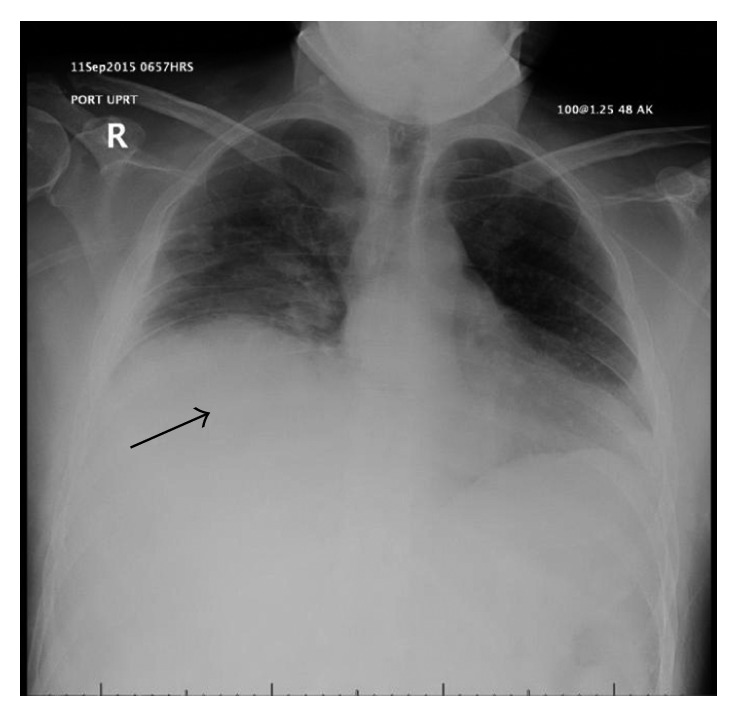
Chest X-ray shows a moderate-sized right pleural effusion (arrow), which was later confirmed to be a hemothorax.

**Figure 2:**
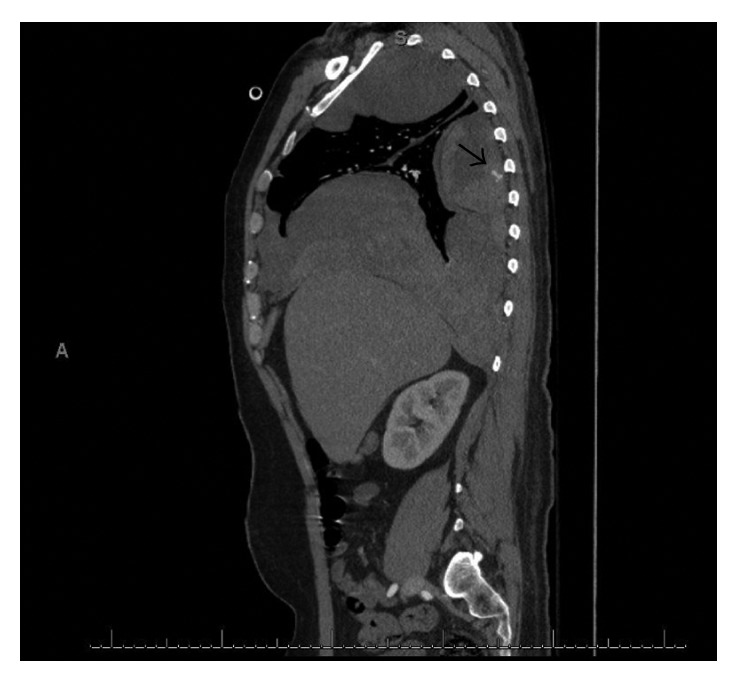
Arterial phase of CT chest scan in sagittal plane shows blushes of contrast (arrow) extending into the hematoma at the level of 7th posterior rib.

**Figure 3:**
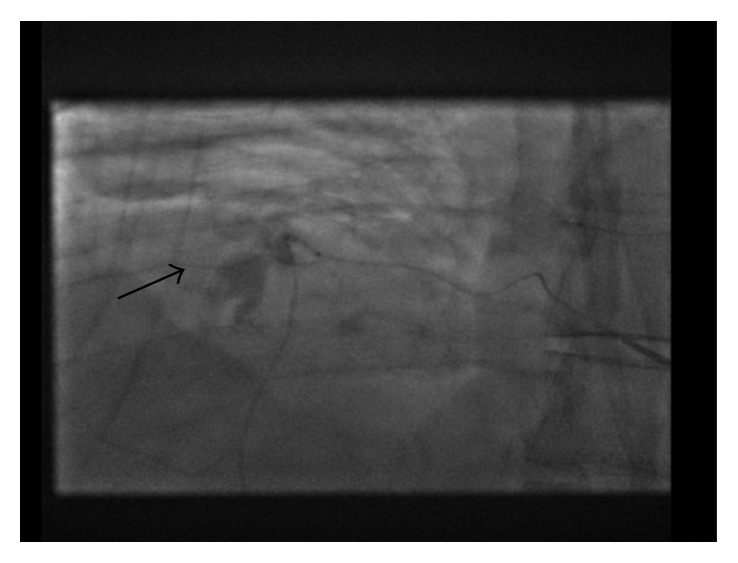
X-ray angiogram of the intercostal arteries shows active contrast extravasation (arrow) from the right T7 intercostal artery.

**Figure 4:**
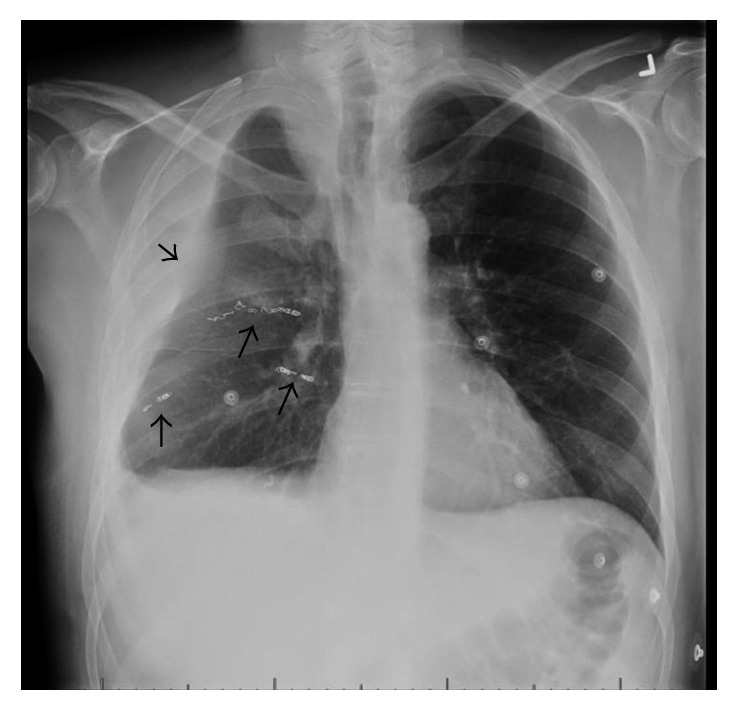
Chest X-ray of the patient on the day of discharge shows metallic coils in the intercostal arteries (arrows) and residual volume loss in the right lung (arrow head) following thoracotomy.

Seventeen days into hospital admission, the patient complained of increased right chest pain and shortness of breath. Chest X-ray showed a moderate-sized pleural effusion in the right lung (Figure 1). His hemoglobin dropped overnight from 108 g/L to 92 g/L with no overt signs of bleeding. CT chest (Figure 2) and subsequent intercostal arteriogram of T6-T12 intercostal arteries showed active contrast extravasations from the right T7 intercostal artery (Figure 3), right T8 intercostal artery, and small branch arising from right T10 intercostal artery. This appeared to be ruptured mycotic aneurysms involving intercostal vessels. Embolization of these vessels was successfully performed using microcoils. He then underwent thoracotomy to evacuate large amounts of blood and clot from the right lung. The patient then completed his course of antimicrobial therapy in the hospital with no further complications (Figure 4).

## 3. Discussion

IVDU is often associated with right-sided IE [2]. Particular matters found in illicit drugs are injected into the vein and travel first to the right side of the heart and cause endothelial damage to the tricuspid valve. Sometimes small particles can cross the pulmonary capillaries and damage the mitral and aortic valves causing left-sided IE [3]. IVDU can further increase the risk of IE since repetitive IVDU can lead to subclinical valvular damage, bacteria and fungi found on skin or in the drug can be introduced to systemic circulation through intravenous injection, and the use of cocaine is associated with increased risk of IE, likely due to vasospasm leading to skin and tissue damage [4].

Systemic septic emboli can be seen in 25–50% of patients with infective endocarditis (IE) [5]. Septic emboli can give rise to mycotic aneurysm, which is a localized dilation of the artery due to obstruction of the vessel wall by infection, and are reported in 1–5% of patients with IE. The majority of mycotic aneurysms in IE are found in the cerebral circulation, followed by the visceral arteries and arteries of the upper and lower extremities [5]. Rare cases of systemic extracranial mycotic aneurysms due to IE have been reported in the popliteal, ulnar, humeral, hepatic, pulmonary, and coronary arteries [6]. The patient in our case did not exhibit symptoms of systemic emboli in these more common embolic areas.

Mycotic aneurysms can be caused by conditions other than septic embolization from IE. Arterial injury can lead to direct inoculation of bacteria into the arterial wall, which can be seen in bacteremia, trauma, catheter-based procedures, or the IVDU population. Numerous cases of contiguous spread of local infection have also been reported, mostly in postoperative patients [6].

Etiology of mycotic aneurysm of the intercostal artery follows a similar pattern. The majority of mycotic aneurysms of the intercostal artery are associated with coarctation of the aorta, possibly due to altered flow dynamics, and can be seen in up to 40% of these patients [7]. Trauma, interventional procedures, bacteremia, and local infections have also been reported as a cause [8].

## 4. Conclusion

This is a case of hemothorax due to ruptured mycotic aneurysms of the intercostal arteries in a 40-year-old male with MRSA IE. Our case report highlights the possibility of ruptured intercostal mycotic aneurysm in patients with IE and hemothorax and reminds physicians to be alert of this rare but potentially fatal complication.
